# Impact of COVID-19 on recorded blood pressure screening and hypertension management in England: an analysis of monthly changes in the quality and outcomes framework indicators in OpenSAFELY

**DOI:** 10.1136/openhrt-2024-002732

**Published:** 2024-08-30

**Authors:** Milan Wiedemann, Victoria Speed, Christine Cunningham, Rose Higgins, Helen J Curtis, Colm Andrews, Louis Fisher, Lisa Hopcroft, Christopher T Rentsch, Viyaasan Mahalingasivam, Laurie Tomlinson, Caroline Morton, Miriam Samuel, Amelia Green, Christopher Wood, Andrew D Brown, Jon Massey, Caroline Walters, Rebecca M Smith, Peter Inglesby, David Evans, Steven Maude, Iain Dillingham, Alex J Walker, Jessica Morley, Amir Mehrkar, Seb Bacon, Chris Bates, Jonathan Cockburn, John Parry, Frank Hester, Richard J McManus, Ben Goldacre, Brian MacKenna

**Affiliations:** 1 Nuffield Department of Primary Care Health Sciences, University of Oxford, Oxford, UK; 2 Faculty of Epidemiology and Population Health, London School of Hygiene & Tropical Medicine, London, UK; 3 Epidemiology and Population Health, London School of Hygiene and Tropical Medicine Faculty of Epidemiology and Population Health, London, UK; 4 Wolfson Institute of Population Health, Queen Mary University of London, London, UK; 5 The DataLab, Nuffield Department of Primary Care Health Sciences, University of Oxford, Oxford, UK; 6 TPP, Leeds, UK

**Keywords:** COVID-19, Hypertension, Quality of Health Care

## Abstract

**Background:**

The COVID-19 pandemic disrupted cardiovascular disease management in primary care in England.

**Objective:**

To describe the impact of the pandemic on blood pressure screening and hypertension management based on a national quality of care scheme (Quality and Outcomes Framework, QOF) across key demographic, regional and clinical subgroups.

**Methods:**

With NHS England approval, a population-based cohort study was conducted using OpenSAFELY-TPP on 25.2 million NHS patients registered at general practices (March 2019 to March 2023). We examined monthly changes in recorded blood pressure screening in the preceding 5 years in patients aged ≥45 years and recorded the hypertension prevalence and the percentage of patients treated to target (≤140/90 mmHg for patients aged ≤79 years and ≤150/90 mmHg for patients aged ≥80 years) in the preceding 12 months.

**Results:**

The percentage of patients aged ≥45 years who had blood pressure screening recorded in the preceding 5 years decreased from 90% (March 2019) to 85% (March 2023). Recorded hypertension prevalence was relatively stable at 15% throughout the study period. The percentage of patients with a record of hypertension treated to target in the preceding 12 months reduced from a maximum of 71% (March 2020) to a minimum of 47% (February 2021) in patients aged ≤79 years and from 85% (March 2020) to a minimum of 58% (February 2021) in patients aged ≥80 years before recovery. Blood pressure screening rates in the preceding 5 years remained stable in older people, patients with recorded learning disability or care home status.

**Conclusions:**

The pandemic substantially disrupted hypertension management QOF indicators, which is likely attributable to general reductions of blood pressure measurement including screening. OpenSAFELY can be used to continuously monitor changes in national quality-of-care schemes to identify changes in key clinical subgroups early and support prioritisation of recovery from care disrupted by COVID-19.

**WHAT IS ALREADY KNOWN ON THIS TOPIC**

- The COVID-19 pandemic disrupted cardiovascular disease management in primary care in England.

**WHAT THIS STUDY ADDS**

- We have reported monthly QOF outcomes for blood pressure monitoring and hypertension control across key demographic, regional and clinical subgroups during the COVID-19 pandemic.
- We have translated the QOF business rules for blood pressure monitoring and hypertension control from text descriptions into a reusable and modifiable code.

**HOW THIS STUDY MIGHT AFFECT RESEARCH, PRACTICE OR POLICY**

- The OpenSAFELY platform could be used to report current and future indicators of clinical care and to identify health inequalities among regional, demographic or clinical sub-populations in near real-time.

## Introduction

 The COVID-19 pandemic disrupted healthcare services globally.^1^ Cardiovascular disease (CVD) management in primary care in England was impacted^2^,^4^ with an estimated 2175 non-COVID excess deaths attributed to hypertensive diseases between March 2020 and December 2021.^5^ CVD is associated with a higher risk of morbidity and mortality from COVID-19, emphasising the importance of maintaining good routine care.^6 7^ High blood pressure is the leading risk factor for CVD^8^ and one of the top three risk factors for global disease burden.^9^ Since 1994, there has been an improvement in the management of high blood pressure in England^10^ and a reduction of the negative impact of social deprivation on blood pressure management.^11^ Delayed management of hypertension is associated with worse clinical outcomes, for example, stroke.^12^ Recent results from annual national audits of England’s population on CVD have also suggested that blood pressure management was disrupted by the pandemic.^13^

In 2004, the Quality and Outcomes Framework (QOF) was introduced in England as one of the largest initiatives worldwide to improve the quality of care in general practice. General practitioners (GPs) and their staff are measured on indicators of good clinical care and receive financial incentives based on their achievement of certain thresholds.^14 15^ To monitor the indicators and thresholds, NHS Digital publishes text descriptions of analytic rules and logic, commonly referred to as ‘*business rules’*, which are taken by software providers and implemented in GP electronic health record systems. GPs can review their delivery of care against these rules and indicators for their practice throughout the financial year; however, national data for all practices are only available annually. At the end of every NHS financial year on March 31, NHS Digital calculates each practice’s achievement against set thresholds for individual indicators. Between 1 April 2020 and 31 March 2023, amendments were made to QOF, and some preventative indicators were suspended, including hypertension management, to support the COVID-19 response and support roll out of the national COVID-19 vaccination programme.^16 17^

OpenSAFELY is a secure analytics platform for electronic patient records built by our group on behalf of NHS England to deliver urgent academic^6^ and operational research^2^ during the pandemic. Using OpenSAFELY-TPP, we therefore aimed to describe trends and variations in these indicators before and during the COVID-19 pandemic and assess recovery of the indicators to pre-pandemic levels across key clinical and demographic subgroups.

## Methods

### Data source

Primary care records managed by the GP software provider TPP were accessed through OpenSAFELY (https://opensafely.org). OpenSAFELY is a secure analytics platform for electronic patient records built by our group with the approval of NHS England to deliver urgent academic^6^ and operational NHS service research^2^ on the direct and indirect impacts of the pandemic.

OpenSAFELY provides a secure software interface allowing the analysis of pseudonymised primary care patient records from England in near real-time within the EHR vendor’s highly secure data centre, avoiding the need for large volumes of potentially disclosive pseudonymised patient data to be transferred off-site. The dataset analysed within OpenSAFELY is based on 25 million people currently registered with GP surgeries using TPP SystmOne software. It includes pseudonymised data such as coded diagnoses, medications and physiological parameters. No free text data are included. Further details on our information governance and ethics can be found in the in the online supplemental file 1.

### Study design and population

We conducted a retrospective cohort study from March 2019 to March 2023 using primary care EHR data from all GP practices in England supplied by the EHR vendor TPP, a cohort that is broadly representative of the population in England.^18^ Following the QOF business rules, we included all patients who were alive and registered with an OpenSAFELY-TPP practice for the QOF hypertension prevalence and management indicators (HYP001, HYP003, HYP007). For the QOF blood pressure screening indicator (BP002), we included only those aged ≥45 years.

### Implementation of QOF business rules in analytic code

QOF indicators for blood pressure screening in the preceding 5 years (BP002), hypertension register (HYP001) and hypertension management in the preceding 12 months (HYP003, HYP007) were specified in analytic code replicating the QOF business rules for 2021/22 [(Version 46, 19](Version 46)^19^ using the OpenSAFELY framework (table 1). All QOF indicators are formed by specifying rules and logic, which determine aggregate counts of patients. Percentages are then calculated using numerator and denominator pairs. In addition to QOF indicators, we also applied the same clinical rules for blood pressure screening (BP002) to all patients with a record of an unresolved hypertension diagnosis using a 12-month lookback period to match the timeframe used in the hypertension management indicators (HYP003 and HYP007).

**Table 1:** Descriptions of the Quality and Outcomes Framework indicators for blood pressure (BP) and hypertension (HYP)

Indicator	Domain/category	Indicator description	Population of interest
BP002	Public health	The percentage of patients aged 45 years or over who have a record of blood pressure **in the preceding 5 years**.	All registered patients aged ≥45 years
HYP001*	Clinical/records	The contractor establishes and maintains a register of patients with established hypertension.	All registered patients
HYP003†	Clinical/ongoing management	The percentage of patients aged 79 years or below, with hypertension, in whom the last blood pressure reading (measured **in the preceding 12 months**) is 140/90 mmHg or less.	Hypertension register (HYP001*)
HYP007[Table-fn T1_FN2]	Clinical/ongoing management	The percentage of patients aged 80 years or over, with hypertension, in whom the last blood pressure reading (measured **in the preceding 12 months**) is 150/90 mmHg or less.	Hypertension register (HYP001*)

* Indicator HYP001 refers to the hypertension register (HYP_REG) which is defined as ‘Patients with an unresolved diagnosis of hypertension’.

† For the purpose of this analysis, this is the treatment target. The population that meets the blood pressure target will be described as *treated to target*. More details on the specific selection and exclusion rules and the codelists are available in the online supplemental Tables 1 - 5online supplemental tables 1–5.

Higher indicator percentages represent a higher percentage of patients receiving indicated clinical care. Patients can be excluded from the denominator according to QOF rules, such as those who declined treatment (for more details, see online supplemental tables 1–4. For the two hypertension control indicators (HYP003 and HYP007), patients with hypertension who did not have their blood pressure recorded in the last year are counted as not being treated to target as per the QOF business rules.

Data were analysed for each month between 1 March 2019 and 31 March 2023 covering five financial years. Each monthly cohort replicated the yearly reporting of each QOF business rule. Thus, the data presented for each March in this study align with the reporting period of the corresponding annual QOF reports published by NHS Digital.

### Monthly changes in QOF indicators across demographic, regional and clinical subgroups

Trends and variations in QOF indicators were reported across demographic (10-year age bands, sex, ethnicity in 5 and 16 categories), regional (practice level deciles, Indices of Multiple Deprivation quintiles derived from patient’s postcode at lower super output area, region) and key clinical subgroups (record of learning disability and care home status) highlighted in the NHS long-term plan as priority groups.^20^

### Software and reproducibility

Data management and analyses were performed using the OpenSAFELY software libraries using with Python (Version 3.8.10) and R (Version 4.0.2). Codes replicating the QOF business rules are available at https://github.com/opensafely/hypertension-sro https://github.com/opensafely/hypertension-sro and https://github.com/opensafely/blood-pressure https://github.com/opensafely/blood-pressure-sro alongside all analytic code and codelists. The GitHub repository https://github.com/opensafely/qof-utilities https://github.com/opensafely/qof-utilities contains reusable code developed for implementing QOF rules in OpenSAFELY.

### Patient and public involvement

For transparency purposes, we have developed a public website (https://opensafely.org/) that provides a detailed description of the platform in language suitable for a lay audience; we have participated in two citizen juries exploring public trust in OpenSAFELY.^21^ To ensure the patient voice is represented, we are working closely with appropriate medical research charities; however, there was no patient or public involvement in this specific research question.

## Results

### Calculating monthly trends in QOF indicators

Detailed demographic characteristics of all patients considered for the blood pressure (n=11 195 670) and hypertension indicators (n=25 287 730) during the reporting period of the NHS financial year 21/22 are presented in table 2.

**Table 2:** Cohort description for patients included in the blood pressure and hypertension Quality and Outcomes Framework indicators in OpenSAFELY-TPP during the NHS financial year 21/22

	BP002 (Age>=45)Public health domain			HYP001 (total population)clinical domain			HYP003 (age ≤79)vlinical domain			HYP007 (Age ≥80)clinical domain		
	Numerator	Denominator	Receiving indicated care	Register	List size	Prevalence	Numerator	Denominator	Receiving indicated care	Numerator	Denominator	Receiving indicated care
Population												
	9 587 610	11 195 670	85.6%	3 672 870	25 287 730	14.5%	1 607 520	2 664 840	60.3%	554 240	764 960	72.5%
Sex												
Female	5 115 540	5 736 940	89.2%	1 848 910	12 621 240	14.7%	782 460	1 268 720	61.7%	326 560	463 200	70.5%
Male	4 472 070	5 458 730	82.0%	1 823 960	12 666 500	14.4%	825 060	1 396 110	59.1%	227 680	301 760	75.5%
Age band												
0–19	–	–	–	2180	5 547 410	0.04%	1010	1780	56.7%	–	–	–
20–29	–	–	–	11 400	3 139 340	0.4%	4790	9120	52.5%	–	–	–
30–39	–	–	–	60 680	3 629 760	1.7%	25 760	50 520	51.0%	–	–	–
40–49	1 124 490	1 548 870	72.6%	220 760	3 246 940	6.8%	99 700	191 390	52.1%	–	–	–
50–59	2 751 360	3 415 030	80.6%	615 040	3 458 290	17.8%	308 270	555 420	55.5%	–	–	–
60–69	2 407 340	2 752 690	87.5%	887 520	2 774 540	32.0%	506 800	828 700	61.2%	–	–	–
70–79	2 079 410	2 215 190	93.9%	1 079 680	2 224 430	48.5%	661 200	1 027 890	64.3%	–	–	–
80+	1 225 010	1 263 890	96.9%	795 620	1 267 020	62.8%	–	–	–	554 240	764 960	72.5%
Ethnicity												
Asian or Asian British												
Any other Asian background	106 280	126 810	83.8%	39 230	412 650	9.5%	21 570	33 220	64.9%	2250	3080	73.1%
Bangladeshi	27 800	30 490	91.2%	11 860	124 500	9.5%	6900	10 060	68.6%	570	820	69.5%
Indian	209 490	239 700	87.4%	91 540	710 370	12.9%	47 230	74 360	63.5%	8100	11 090	73.0%
Pakistani	123 280	136 790	90.1%	48 560	513 040	9.5%	25 980	40 040	64.9%	3530	4900	72.0%
Black or Black British												
African	90 470	108 040	83.7%	40 280	385 090	10.5%	18 710	34 270	54.6%	1000	1620	61.7%
Any other Black background	29 650	34 290	86.5%	11 700	103 910	11.3%	5340	9760	54.7%	570	840	67.7%
Caribbean	55 000	61 340	89.7%	27 660	115 680	23.9%	11 410	19 970	57.1%	4160	5900	70.5%
Mixed												
Any other mixed background	22 820	28 270	80.7%	7480	141 140	5.3%	3600	6150	58.5%	450	630	71.4%
White and Asian	12 650	15 320	82.6%	3970	81 280	4.9%	1980	3270	60.6%	240	340	70.6%
White and Black African	13 620	16 600	82.1%	5660	70 890	8.0%	2590	4770	54.3%	150	250	60.0%
White and Black Caribbean	15 690	17 990	87.2%	6710	84 870	8.0%	2840	5160	55.0%	690	970	71.1%
Other ethnic groups												
Any other ethnic group	69 430	89 460	77.6%	21 420	330 160	6.5%	10 320	17 140	60.2%	1610	2210	72.9%
Chinese	31 660	43 510	72.8%	9030	182 960	5.0%	4520	7100	63.7%	720	1030	69.9%
White												
Any other White background	589 110	755 220	78.0%	220 030	2 320 120	9.5%	92 240	163 970	56.3%	26 690	37 150	71.84%
British	6 951 180	7 855 620	88.5%	2 607 310	14 450 100	18.0%	1 151 190	1 881 440	61.2%	409 870	559 430	73.27%
Irish	58 040	66 660	87.1%	22 080	112 460	19.6%	8790	14 600	60.2%	4360	6020	72.43%
(Missing)	1 181 420	1 569 530	75.3%	498 350	5 148 500	9.7%	192 300	339 570	56.6%	89 300	128 680	69.40%
IMD												
1 - Most deprived	1 541 010	1 793 920	85.9%	634 660	4 993 420	12.7%	298 170	486 270	61.3%	74 240	102 320	72.6%
2	1 728 400	2 015 760	85.7%	688 070	4 885 930	14.1%	307 680	509 890	60.3%	94 540	130 790	72.3%
3	2 078 700	2 425 360	85.7%	799 060	5 203 780	15.4%	344 130	573 440	60.0%	126 130	173 430	72.7%
4	2 062 080	2 410 990	85.5%	769 580	4 892 080	15.7%	327 280	545 930	60.0%	126 760	175 140	72.4%
5 - Least deprived	1 977 820	2 313 780	85.5%	709 070	4 503 140	15.8%	297 250	496 010	59.9%	123 440	170 860	72.3%
(Missing)	199 590	235 860	84.6%	72 440	809 380	9.0%	33 000	53 300	61.9%	9140	12 420	73.6%
Region												
East	2 190 210	2 552 650	85.8%	828 930	5 785 610	14.3%	349 120	596 850	58.5%	126 920	178 030	71.3%
East Midlands	1 689 470	1 966 690	85.9%	658 520	4 391 220	15.0%	295 020	481 870	61.2%	97 480	132 650	73.5%
London	467 680	581 050	80.5%	174 910	1 798 910	9.7%	87 120	138 270	63.0%	19 930	27 610	72.2%
North East	444 680	516 940	86.0%	175 280	1 176 200	14.9%	81 040	129 640	62.5%	26 460	35 520	74.5%
North West	896 960	1 034 710	86.7%	355 940	2 185 660	16.3%	172 190	263 810	65.3%	51 580	69 400	74.3%
South East	657 640	775 260	84.8%	238 870	1 647 190	14.5%	91 050	167 610	54.3%	37 710	55 340	68.1%
South West	1 484 430	1 733 970	85.6%	547 440	3 508 660	15.6%	219 820	375 880	58.5%	94 190	129 810	72.6%
West Midlands	355 330	412 660	86.1%	144 560	1 041 080	13.9%	61 510	108 300	56.8%	20 180	28 890	69.9%
Yorkshire and The Humber	1 374 980	1 593 520	86.3%	538 010	3 666 360	14.7%	246 450	397 970	61.9%	78 570	106 420	73.8%
Care home status												
No	9 443 510	11 049 600	85.5%	3 617 810	25 175 090	14.37%	1 599 120	2 652 850	60.3%	526 350	727 500	72.4%
Yes	144 100	146 060	98.7%	55 060	112 640	48.88%	8390	11 980	70.0%	27 890	37 460	74.5%
Record or learning disability												
No	9 542 000	11 149 060	85.6%	3 658 810	25 144 720	14.55%	1 598 740	2 652 360	60.3%	553 820	764 410	72.5%
Yes	45 610	46 610	97.6%	14 060	143 010	9.83%	8780	12 480	70.4%	420	550	76.4%

BP002, the percentage of patients aged ≥45 with blood pressure screening in the preceding 5 years; HYP001, hypertension register; HYP003 and HYP007, the percentage of patients aged ≤79 years and ≥80, respectively, diagnosed with hypertension and treated to target in the preceding 12 months.

All counts were rounded to the nearest 10.

### Changes in blood pressure screening and hypertension management rates in the total population

**Figure 1:**
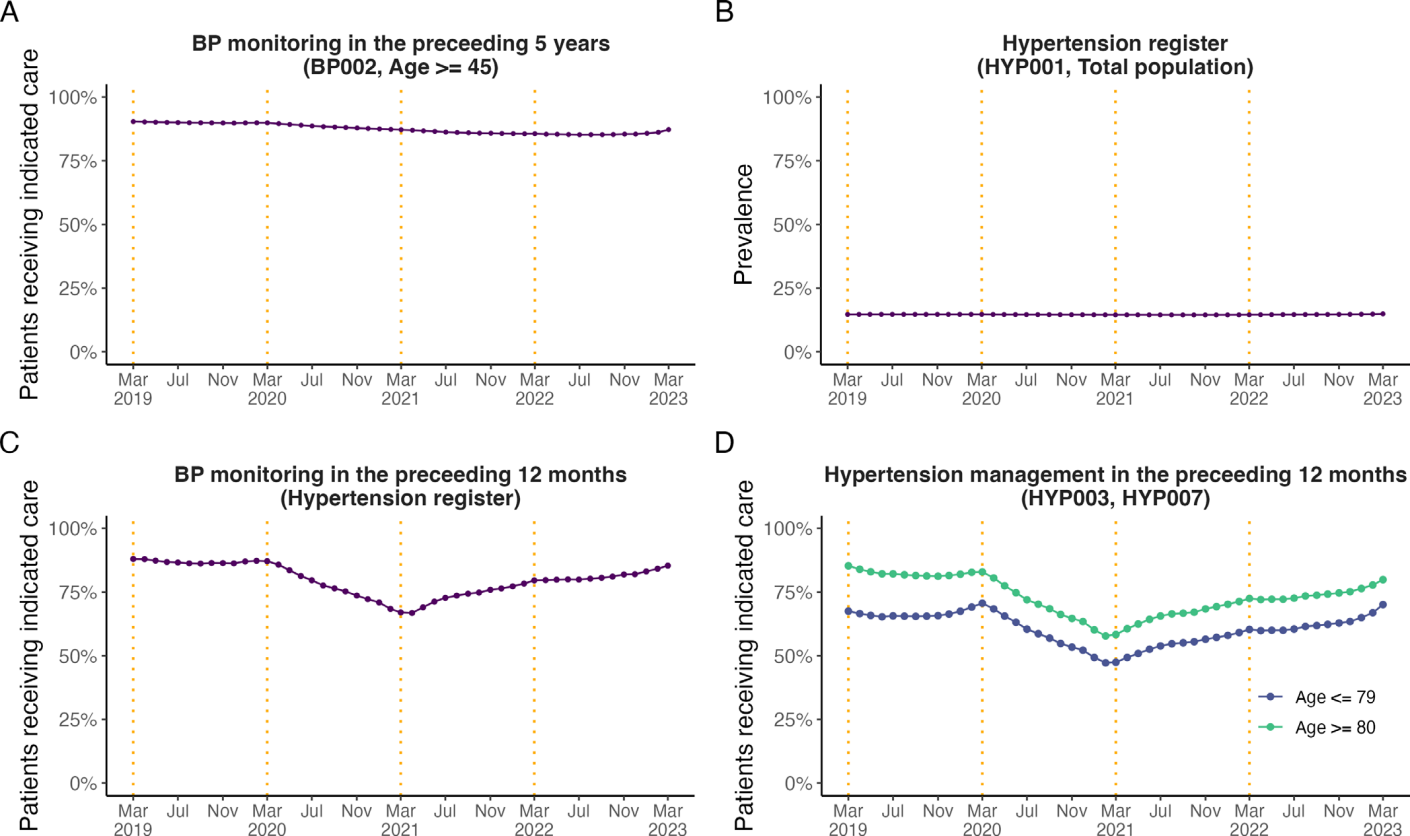
Monthly trends from March 2019 to March 2023 in (**A**) the percentage of patients aged ≥45 years with blood pressure screening in the preceding 5 years (BP002); (**B**) the hypertension register (HYP001); (**C**) the percentage of patients diagnosed with hypertension and recorded blood pressure in the preceding 12 months. Demographic, regional and clinical subgroups for panel C are presented in online supplemental figure 2); (**D**) the percentage of patients diagnosed with hypertension and treated to target (HYP003 and HYP007) in the preceding 12 months. BP, Blood pressure; HYP, Hypertension. The end of the NHS financial years (March) is highlighted with orange dashed vertical lines. Counts of patients in the numerator and denominator pair are presented in online supplemental figures 1C and 2C).

In the total population, the percentage of patients aged ≥45 years (BP002) with recorded blood pressure in the preceding 5 years decreased steadily from its maximum of 90.4% in March 2019 to a minimum of 87.2% in March 2023 (figure 1A).

Recorded hypertension prevalence (HYP001) was relatively stable throughout the entire study period (14.7% in March 2019 to 14.9% in March 2023, figure 1B). Blood pressure screening in the preceding 12 months in patients identified as having hypertension decreased from its peak of 88% in March 2019 to its lowest value of 68% in April 2021 and subsequently improved steadily to 85% in March 2023.

Of those aged ≤79 years identified as having hypertension, the percentage of patients with blood pressure treated to target (HYP003) varied from 67.5% in March 2019 to 70.1% in March 2023, with a peak of 70.6% in March 2020 and a lowest value of 47.2% in February 2021 (figure 1C). Of those aged ≥80 years, the percentage of patients with blood pressure treated to target (HYP007) reduced from 85.3% in March 2019 (the peak value) to 79.9% in March 2023, with a lowest value of 57.8% in February 2021 (figure 1D). For both hypertension management indicators (HYP003 and HYP007) as well as blood pressure screening in patients identified as having hypertension, the results indicated a steady improvement between March 2021 and March 2023.

By March 2023, the differences compared with March 2019 were a 3.1% decrease (90.4% to 87.2%) in patients aged ≥45 years (BP002) with recorded blood pressure in the preceding 5 years, a 0.2% increase (14.7% to 14.9%) in recorded hypertension prevalence (HYP001), a 2.6% increase (67.5% to 70.1%), and a 5.4% decrease (85.3% to 79.9%) in patients identified as having hypertension with blood pressure treated to target aged ≤79 and ≥80 years, respectively (HYP003 and HYP007). Counts of patients in the numerator and denominator are presented in online supplemental figure 1.

### Changes in blood pressure screening and hypertension management rates in demographic, regional and clinical subgroups

#### Subgroups for blood pressure screening in the preceding 5 years in patients aged ≥45 years (BP002)

**Figure 2:**
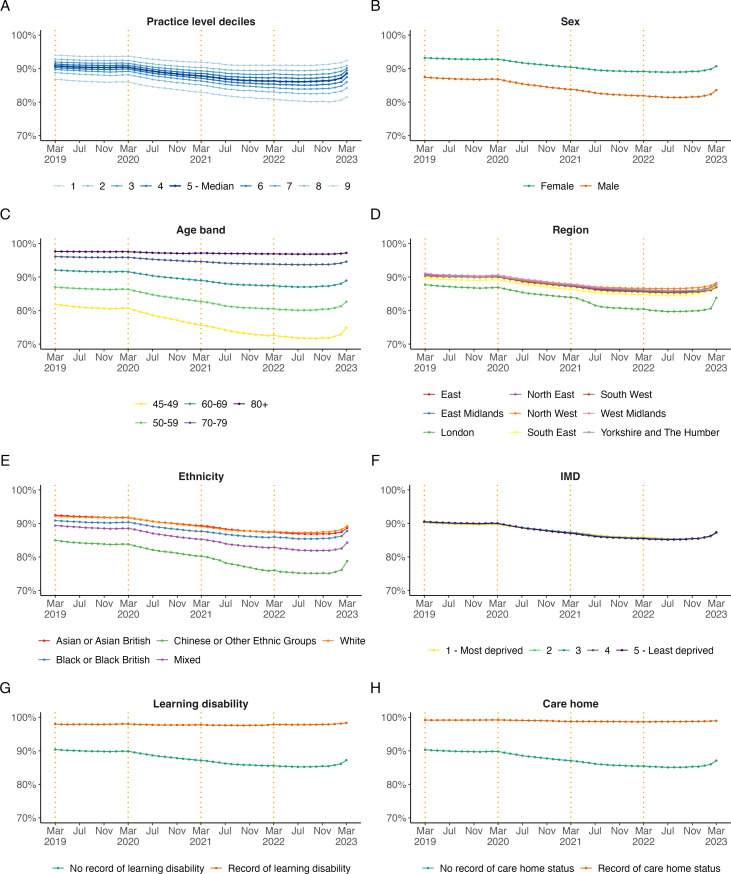
Monthly, unstandardised trends from March 2019 to March 2023 in the percentage of patients aged ≥45 years with recorded blood pressure in the preceding 5 years in (BP002) broken down by (**A**) practice level deciles, (**B**) sex, (**C**) age band, (**D**) region, (**E**) ethnicity, (**F**) IMD, Indices of Multiple Deprivation, (**G**) learning disability and (**H**) care home status for hypertension. The end of the NHS financial years (March) is highlighted with orange dashed vertical lines.

Figure 2 shows the trends in subgroups for blood pressure screening in the preceding 5 years in patients aged ≥45 years (BP002). Preexisting differences in blood pressure screening rates between younger and older age groups increased over the study period, with a reduction in screening observed in younger adults (eg, for age category 45 to 49: 81.9% in March 2019 to 74.9% in March 2023) but not older adults (blood pressure screening was preserved at around 97% in adults aged 80+, figure 2C). Blood pressure screening was also maintained in those with a record of care home status (99.2% in March 2019 to 98.7% in March 2023) or learning disability (98.0% in March 2019 to 98.3% in March 2023, figure 2G,H). From December 2022 to March 2023, results indicate an improvement in recorded blood pressure screening across all demographic and clinical subgroups.

#### Subgroups for hypertension prevalence (HYP001)

**Figure 3:**
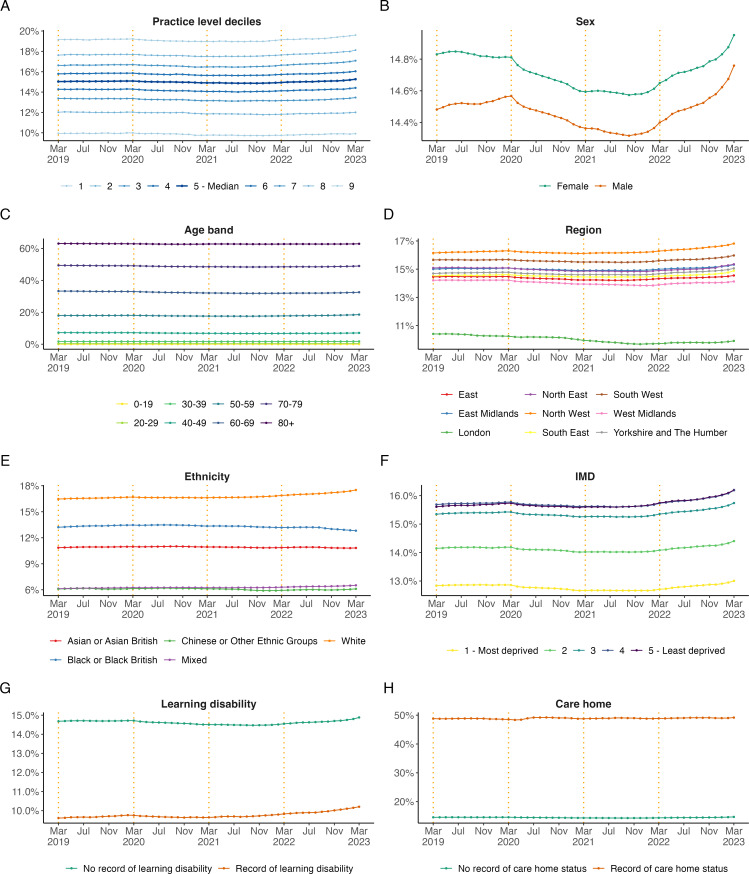
Monthly, unstandardised trends from March 2019 to March 2023 in hypertension prevalence (HYP001) broken down by (**A**) practice level deciles, (**B**) sex, (**C**) age band, (**D**) region, (**E**) ethnicity, (**F**) IMD, Indices of Multiple Deprivation, (**G**) learning disability and (**H**) care home status for hypertension. The end of the NHS financial years (March) is highlighted with orange dashed vertical lines. Note that the range of the y-axis varies by breakdown category to highlight differences between groups.

Grouping by demographic subgroups revealed pre-pandemic differences in hypertension recording (figure 3). In March 2019, the national median by practice was 15.0%; however, this was considerably lower in London (10.4%) and in those with an ethnicity record of ‘Mixed’ (6.1%) or ‘Chinese or Other Ethnic Groups’ (6.9%). Hypertension was also less frequently recorded in those living in the most deprived areas (12.8%) compared with those living in the least deprived areas (15.6%). Hypertension was more often recorded in those living in care homes (48.8%) and less frequently in those with learning difficulties (9.6%). The differences observed at pre-pandemic remained similar throughout the study period across most subgroups (figure 3).

#### Subgroups for hypertension management in the preceding 12 months in patients aged ≤79 (HYP003) and ≥80 (HYP007) years

**Figure 4:**
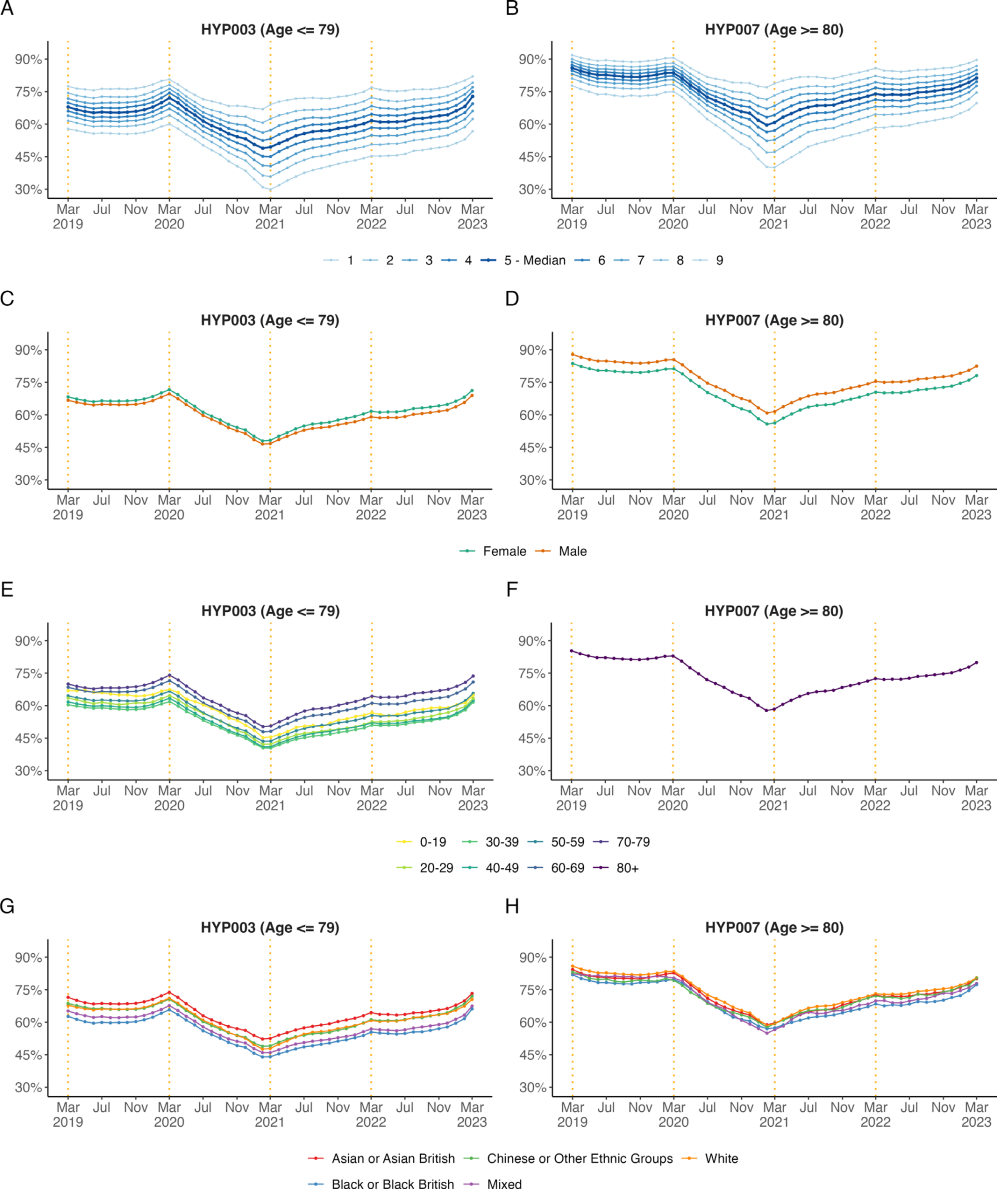
Monthly, unstandardised trends from March 2019 to March 2023 in the percentage of patients diagnosed with hypertension treated to target in the preceding 12 months aged ≤79 years (HYP003) and ≥80 years (HYP007) broken down by (**A, B**) practice level deciles, (**C, D**) sex, (**E, F**) age band and (**G, H**) ethnicity. The end of the NHS financial years (March) is highlighted with orange dashed vertical lines.

**Figure 5:**
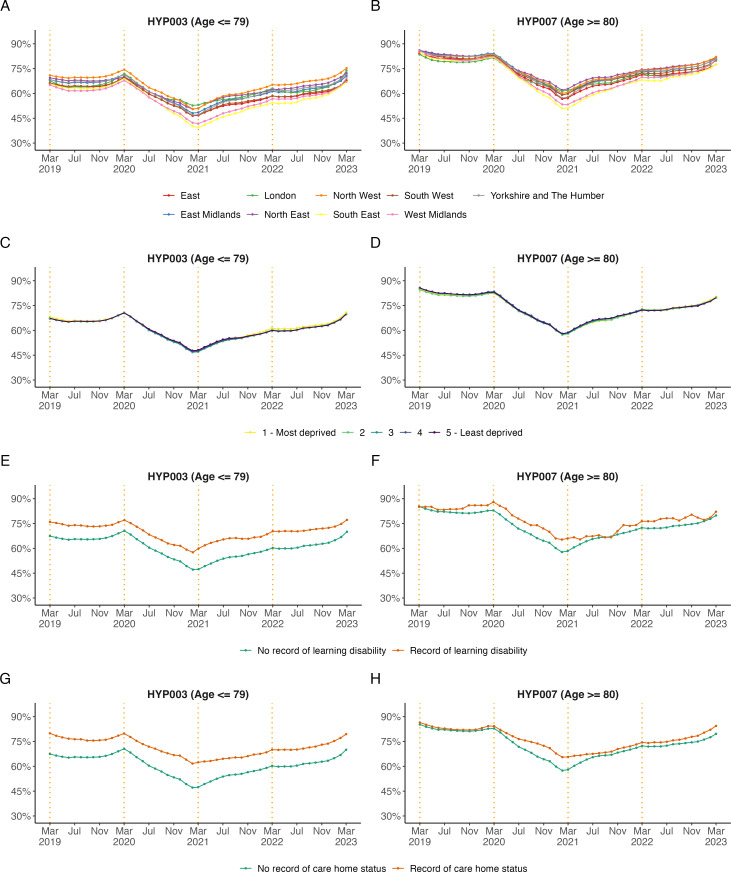
Monthly, unstandardised trends from March 2019 to March 2023 in the percentage of patients diagnosed with hypertension treated to target in the preceding 12 months aged ≤79 years (HYP003) and ≥80 years (HYP007) broken down by (**A, B**) region, (**C, D**) IMD, Indices of Multiple Deprivation, (**E, F**) record of learning disability and (**G, H**) care home status. The end of the NHS financial years (March) is highlighted with orange dashed vertical lines.

Figures4  5 show the trends in subgroups for hypertension management. Preexisting regional differences between subgroups in blood pressure management in patients diagnosed with hypertension aged ≤79 years (HYP003) increased during the study period (figure 4A). In March 2021, the regions South-East and West Midlands had the lowest proportion of patients treated to target, with 39.4% and 41.8%, respectively (figure 5A), compared with the national median by practice of 49.4% and other regions. A similar trend was observed for patients aged ≥80 years (HYP007, figures4B  5B).

Later in the analysis period, between March 2021 and March 2023, a higher proportion of patients in older age groups (60–69 and 70–79 years) were reported as having their hypertension treated to target than younger age groups.

Between March 2020 and March 2021, the proportion of patients in care homes with hypertension treated to target reduced by 17.4% in those aged ≤79 years (HYP003) and by 18.6% in those aged ≥80 years (HYP007). In March 2023, the proportion of patients with hypertension treated to target had nearly returned to pre-pandemic levels.

## Discussion

### Summary

These results suggested that the pandemic had a substantial impact on the percentage of patients in whom hypertension was classified as not treated to target within the preceding 12 months. Our analyses suggest that this may be attributed to a reduction in blood pressure measurement in the preceding 12 months. We did not observe a substantial impact of the pandemic on the public health QOF standard for blood pressure screening, which is assessed within the preceding 5 years, or on the register for hypertension prevalence.

### Strengths and limitations

This study has a range of strengths. The OpenSAFELY-TPP platform runs analyses across the full raw, pseudonymised, dataset for 25.2 million patients at 2540 practices in England using TPP software. In this study, we have reported monthly QOF measures in near real-time.

We acknowledge several limitations in our analyses. Results within regional and demographic subgroups need to be interpreted carefully because we report unstandardised results and did not account for sociodemographic factors (eg, age differences between subgroups). A recent study found that OpenSAFELY-TPP was largely representative of the general population of England in terms of IMD, age, sex, ethnicity and causes of death, although with relative underrepresentation of practices in London.^17^

We have implemented QOF business rules as described in the text description published by NHS Digital; however, we would expect our ascertainment of specific patients to deviate from other sources of QOF data for two reasons: (1) as described above OpenSAFELY-TPP has access to the full raw GP record, which many not be the case for all other sources of QOF data; and (2) when translating information from the QOF business rules we had to make pragmatic decisions to resolve some ambiguity.

Finally, we also note that our data will only include clinical codes for blood pressure recording that were carried out in primary care, by a patient at home and correctly captured in a GP system or in secondary care and returned to GPs as structured data. In this study, where blood pressure results are not recorded or correctly captured within a GP system, individuals were counted as not being treated to target, which is consistent with the QOF business rules. The correct capture of clinical coding is a limitation of all EHR analytic work.

### Findings in context

Blood pressure screening and hypertension management are national priorities^22^ as a modifiable strong risk factor for CVD globally.^23^ Hypertension management has been incentivised by QOF for primary care since 2005, and advances in the detection and management of patients with hypertension have been gradually observed.^10^ This progression was disrupted by the COVID-19 pandemic. In response to the unparalleled pressure on the health service in the pandemic, some QOF indicators were suspended, including hypertension management in order to support prioritisation of clinical workload.^24^ Practices may have rightly deprioritised some care described here to focus on more urgent care needs during the COVID-19 pandemic.

We have reported a prevalence of hypertension that is consistent with the latest available official annual report by NHS Digital covering NHS financial year 2021/22 (see online supplemental table 6) and with the second annual audit from the CVDPREVENT initiative (covering data up to March 2021) in England.^13^ Furthermore, our results are consistent with the most recent quarterly data published by CVDPREVENT including data up to June 2022.^25^ A consistent finding across our work, CVDPREVENT and official QOF publications was the reduction in the proportion of patients with hypertension treated to target in March 2020 and March 2021; however, our study reveals continued recovery of care beyond this period, particularly during the first months of 2023. CVDPREVENT also reported a reduction of 21.4% in the proportion of patients recorded as having their blood pressure treated and meeting the NICE guideline target between March 2020 and March 2021.^13^ It is likely that the combination of the factors contributed to this observation. Those without a blood pressure recording in the preceding 12 months were reported as not having their hypertension treated to target (see online supplemental figure 2). This finding is likely due to a 42% reduction of recorded blood pressure measurement activity between April 2019 and April 2021.^3^ Furthermore, services may have re-prioritised those with poorly controlled blood pressure during the pandemic.

Interestingly, there is a sex- and age-dependent difference in the proportion of patients with hypertension treated to target. The proportion of female patients with hypertension treated to target was consistently greater than male patients aged ≤79 years (figure 4C). However, in patients ≥80 years, this is reversed, with a greater proportion of male patients having their hypertension treated to target (figure 4D). The cause of this trend is beyond the scope of this study, but similar patterns have been observed elsewhere.^26^ Similarly, the unstandardised results suggest that there is a trend towards a lower proportion of black ethnic patients with hypertension treated to target (figure 4G,H); however, this is likely influenced by age differences between ethnicity groups. Variation in hypertension control according to ethnicity has been described widely elsewhere and the cause of which is likely to be multi-faceted.^27^

### Implications for policy and future research

It is possible that the observed results represent prioritisation of other clinical areas, and the results of this study should not be interpreted as criticism of GPs. The issue has already been recognised, and new NHS services were rapidly established in response (eg, NHS Community Pharmacy Blood Pressure Check Service and BP@home Service).^22 28^

In this study, we observed that patients from the most deprived areas had the lowest proportion of patients on the hypertension register (HYP001), and this decreased further during the pandemic (figure 3F). There was also a trend towards a lower proportion of black ethnic patients with hypertension treated to target, which has also been observed prior to the pandemic in other elements of CVD care.^29^ Future research is needed to help understand the reasons for the observed reductions in hypertension management and to further understand the health inequalities to find effective solutions to best address them. The Core20PLUS5, a national NHS England approach to support the reduction of health inequalities at both national and system level, is a new well-placed initiative designed to tackle the management of high blood pressure in these important patient groups.

The OpenSAFELY platform could be used to analyse NHS England, National Institute of Clinical Excellence and Care Quality Commission on current indicators of clinical care and has the technical ability to prototype new ones. The additional demographic and clinical data securely accessible through the OpenSAFELY tools also has a number of advantages to current published reports of QOF. It can be used to identify health inequalities among regional, demographic or clinical sub-populations (eg, ethnicity or record of learning disability) in near real-time for new policies and clinical recommendations. Further, all code used in OpenSAFELY is reusable and modifiable and is available under open source licences, which provides opportunities for a more transparent future for operational analysis in the NHS and research using EHR data.

## Conclusion

Although hypertension management indicators were disrupted substantially during the pandemic, this can likely be attributed to a general reduction of blood pressure measurement. Reassuringly, hypertension management indicators have been improving steadily since March 2021 and are now approaching those seen pre-pandemic. While resources were stretched during the pandemic, blood pressure screening was prioritised by GPs in older age groups and patients with a record of learning disability or care home status. OpenSAFELY can be used to continuously monitor monthly changes in quality of care indicators to identify significant changes in key clinical subgroups early.

## supplementary material

10.1136/openhrt-2024-002732 online supplemental file 1

## Data Availability

All data were linked, stored and analysed securely within the OpenSAFELY platform (https://opensafely.org/). Data include pseudonymised data such as coded diagnoses, drugs and physiological parameters. No free text data were included. All code is shared openly for review and reuse under MIT open license (https://github.com/opensafely/blood-pressure-sro and https://github.com/opensafely/hypertension-sro). Detailed pseudonymised patient data are potentially re-identifiable and therefore not shared.
