# Supplementary material for: Impact of COVID-19 on recorded blood pressure screening and hypertension management in England: an analysis of monthly changes in the quality and outcomes framework indicators in OpenSAFELY
Source: Open Heart. 2024 Aug 30;11(2):e002732. doi: 10.1136/openhrt-2024-002732 (PMC11664366; doi:10.1136/openhrt-2024-002732)
Supplement: online supplemental file 1 [file openhrt-11-2-s001.pdf]

Supplemental Material

A. Denominator and numerator rules

Table 1. Descriptions of denominator and numerator rules for QOF indicator BP002 (Version 46). Note that the denominator rule 2 and numerator rule 1 select conditions are identical, but describe different actions for cases that do not meet the conditions (next rule vs reject).

	Number	Description
**Denominator**	1	Reject patients from the specified population who are aged less than 45 years old. Pass all remaining patients to the next rule.
	2	Select patients passed to this rule who had their blood pressure recorded in the 5 year period leading up to and including the payment period end date. Pass all remaining patients to the next rule.
	3	Reject patients passed to this rule chose not to have their blood pressure recorded in the 5 year period leading up to and including the payment period end date. Pass all remaining patients to the next rule.
	4	Reject patients passed to this rule who registered with the GP practice in the 3 month period leading up to and including the payment period end date. Select the remaining patients.
**Numerator**	1	Select patients from the denominator who had their blood pressure recorded in the 5 year period leading up to and including the payment period end date. Reject the remaining patients.

Table 2. Descriptions of denominator and numerator rules for QOF indicator HYP_REG / HYP001 (Version 46).

Number	Description
1	Select patients from the specified population who have a diagnosis of hypertension which has not been subsequently resolved. Reject the remaining patients.

Table 3. Descriptions of denominator and numerator rules for QOF indicator HYP003 (Version 46). Note that the denominator rule 2 and numerator rule 1 select conditions are identical, but describe different actions for cases that do not meet the conditions (next rule vs reject).

	Number	Description
**Denominator**	1	Reject patients from the specified population who are aged greater than 79 years old. Pass all remaining patients to the next rule.
	2	Select patients passed to this rule who meet all of the criteria below:<br>● Systolic blood pressure value was 140 mmHg or less.<br>● Diastolic blood pressure value was 90 mmHg or less.<br>● Most recent blood pressure recording was in the 12 months leading up to and including the payment period end date.<br>Pass all remaining patients to the next rule.
	3	Reject patients passed to this rule who are receiving maximal blood pressure therapy in the 12 months leading up to and including the payment period end date. Pass all remaining patients to the next rule.
	4	Reject patients passed to this rule for whom hypertension quality indicator care was unsuitable in the 12 months leading up to and including the payment period end date. Pass all remaining patients to the next rule.
	5	Reject patients passed to this rule who chose not to have their blood pressure recorded in the 12 months leading up to and including the payment period end date. Pass all remaining patients to the next rule.
	6	Reject patients passed to this rule who chose not to receive hypertension quality indicator care in the 12 months leading up to and including the payment period end date. Pass all remaining patients to the next rule.
	7	Reject patients passed to this rule who meet either of the criteria below:<br>● Latest blood pressure reading in the 12 months leading up to and including the payment period end date was above target levels (systolic value of over 140 mmHg and/or a diastolic value of over 90 mmHg), and was followed by two invitations for hypertension monitoring.<br>● Received two invitations for hypertension monitoring and had no blood pressure recordings during the 12 months leading up to and including the achievement date.<br>Pass all remaining patients to the next rule.
	8	Reject patients passed to this rule whose earliest hypertension diagnosis was in the 9 months leading up to and including the payment period end date. Pass all remaining patients to the next rule.
	9	Reject patients passed to this rule who were recently registered at the practice (patient registered in the 9 month period leading up to and including the payment period end date). Select the remaining patients.
**Numerator**	1	Select patients from the denominator who meet all of the criteria below:<br>● Systolic blood pressure value was 140 mmHg or less.<br>● Diastolic blood pressure value was 90 mmHg or less.<br>● Most recent blood pressure recording was in the 12 months up to and including the payment period end date.<br>Reject the remaining patients.

Table 4. Descriptions of denominator and numerator rules for QOF indicator HYP007 (Version 46). Note that the denominator rule number 2 and numerator rule number 1 select conditions are identical, but describe different actions for cases that do not meet the conditions (next rule vs reject).

	Number	Description
**Denominator**	1	Reject patients from the specified population who are aged less than 80 years old. Pass all remaining patients to the next rule.
	2	Select patients passed to this rule who meet all of the criteria below:<br>● Systolic blood pressure value was 150 mmHg or less.<br>● Diastolic blood pressure value was 90 mmHg or less.<br>● Most recent blood pressure recording was in the 12 months leading up to and including the payment period end date.<br>Pass all remaining patients to the next rule.
	3	Reject patients passed to this rule who are receiving maximal blood pressure therapy in the 12 months leading up to and including the payment period end date. Pass all remaining patients to the next rule.
	4	Reject patients passed to this rule for whom hypertension quality indicator care was unsuitable in the 12 months leading up to and including the payment period end date. Pass all remaining patients to the next rule.
	5	Reject patients passed to this rule who chose not to have their blood pressure recorded in the 12 months leading up to and including the payment period end date. Pass all remaining patients to the next rule.
	6	Reject patients passed to this rule who chose not to receive hypertension quality indicator care in the 12 months leading up to and including the payment period end date. Pass all remaining patients to the next rule.
	7	Reject patients passed to this rule who meet either of the criteria below:<br>● Latest blood pressure reading in the 12 months leading up to and including the payment period end date was above target levels (systolic value of over 150 mmHg and/or a diastolic value of over 90 mmHg), and was followed by two invitations for hypertension monitoring.<br>● Received two invitations for hypertension monitoring and had no blood pressure recordings during the 12 months leading up to and including the achievement date.<br>Pass all remaining patients to the next rule.
	8	Reject patients passed to this rule whose earliest hypertension diagnosis was in the 9 months leading up to and including the payment period end date. Pass all remaining patients to the next rule.
	9	Reject patients passed to this rule who were recently registered at the practice (patient registered in the 9 month period leading up to and including the payment period end date). Select the remaining patients.
**Numerator**	1	Select patients from the denominator who meet all of the criteria below:<br>● Systolic blood pressure value was 140 mmHg or less.<br>● Diastolic blood pressure value was 90 mmHg or less.<br>● Most recent blood pressure recording was in the 12 months up to and including the payment period end date.<br>Reject the remaining patients.

B. Codelists

Table 5. Names and descriptions of clinical code clusters from the NHS Primary Care Domain Reference Portal used to implement the QOF business rules and further codelists.

Cluster name	Description
**Blood pressure**	
BP_COD*	Blood pressure (BP) recording codes
BPDEC_COD	Codes indicating the patient has chosen not to have blood pressure procedure
**Hypertension**	
BP_COD*	Blood pressure (BP) recording codes
BPDEC_COD	Codes indicating the patient has chosen not to have blood pressure procedure
HTMAX_COD	Codes for maximal blood pressure (BP) therapy
HYP_COD	Hypertension diagnosis codes
HYPINVITE_COD	Invite for hypertension care review codes
HYPPCADEC_COD	Codes indicating the patient has chosen not to receive hypertension quality indicator care
HYPPCAPU_COD	Codes for hypertension quality indicator care unsuitable for patient
HYPRES_COD	Hypertension resolved codes
**Breakdown variables**	
LD_COD	Learning disability (LD) codes
ETHNICITY†	Ethnicity codes
CARE_HOME†	NHS care home codes

Note. * The *BP_COD* codelist was manually separated into two codelists: one with all codes referring to systolic blood pressure readings and one with all codes referring to diastolic blood pressure.† These codelists were developed and reviewed using the OpenCodelists platform.

C. Counts of patients in the numerators and denominators of QOF indicators over time

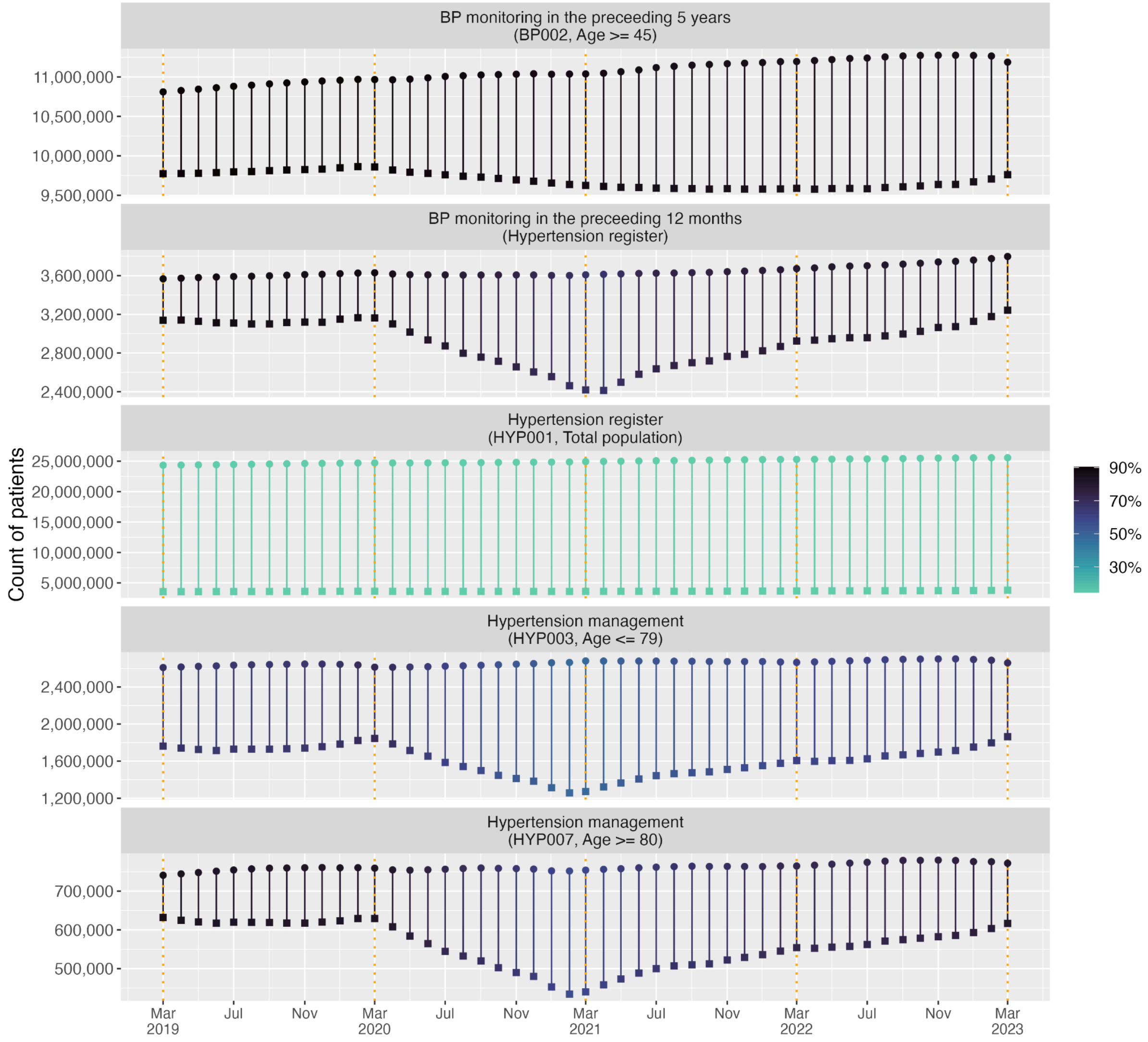

Figure 1. Counts of patients in the numerator and denominator pair for blood pressure and hypertension QOF indicators. The squares indicate the numerator and the circles above represent the denominator. The colour scale indicates the percentage of patients receiving indicated care. The end of the NHS financial years (March) are highlighted with orange dashed vertical lines. Note that the range of the y-axis varies by indicator.

D. Patients diagnosed with hypertension and recorded blood pressure in the preceding 12 months

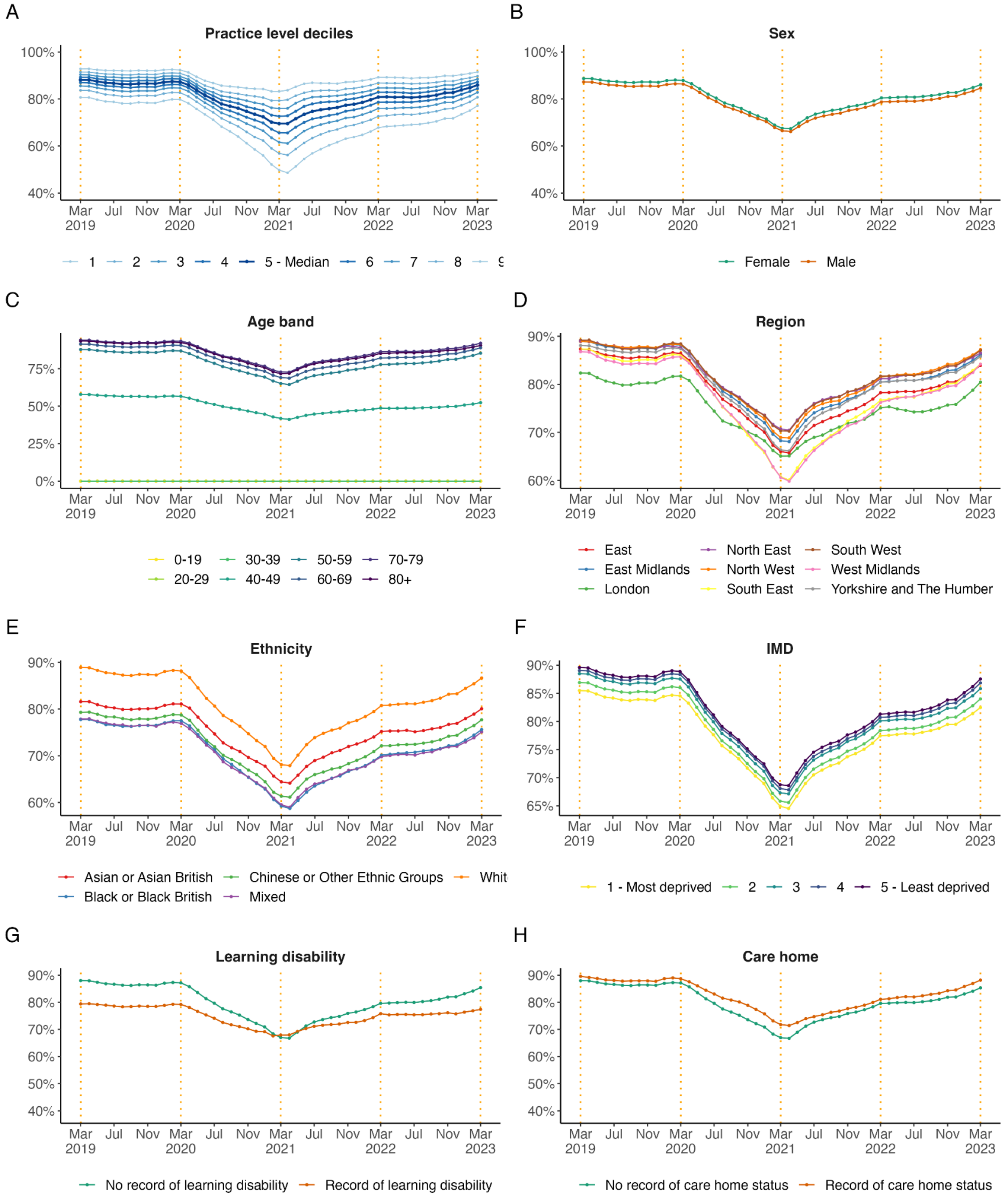

Figure 2. Monthly, unstandardised trends from March 2019 to March 2023 in the percentage of patients with hypertension with recorded blood pressure in the preceding 12 months broken down by (A) practice level deciles, (B) sex, (C) age band, (D) region, (E) ethnicity, (F) IMD = Indices of Multiple Deprivation, (G) learning disability, and (H) care home status for hypertension. The end of the NHS financial years (March) are highlighted with orange dashed vertical lines.

E. Comparison of QOF results in this study with published results by NHS Digital

Table 6. QOF results for the NHS financial year 21/22 in the general population of interest

		NHS FY 21/22		
**Indicator**	**Domain / Category**	**NHSD results**	**This study results[1]**	**Population of interest**
BP002	Public health	85.0%	85.6%	All registered patients aged ≥ 45
HYP001*	Clinical / Records	14.0%	14.4%	All registered patients
HYP003	Clinical / Ongoing management	57.2%	60.4%	Hypertension register (HYP001*)
HYP007	Clinical / Ongoing management	72.2%	72.4%	Hypertension register (HYP001*)

Note. * Indicator HYP001 refers to the hypertension register (HYP_REG) which is defined as 'Patients with an unresolved diagnosis of hypertension'. [1] The results from this study refer to the March 2022 results, which are identical to the time period reported by NHS Digital (NHSD). The results in this study are based on people currently registered with GP surgeries using the TPP SystmOne software.

F. Information governance

NHS England is the data controller for OpenSAFELY-TPP ; [TPP is the data processor]; all study authors using OpenSAFELY have the approval of NHS England. This implementation of OpenSAFELY is hosted within the [TPP environment which is] accredited to the ISO 27001 information security standard and is NHS IG Toolkit compliant [1];

Patient data has been pseudonymised for analysis and linkage using industry standard cryptographic hashing techniques; all pseudonymised datasets transmitted for linkage onto OpenSAFELY are encrypted; access to the platform is via a virtual private network (VPN) connection, restricted to a small group of researchers; the researchers hold contracts with NHS England and only access the platform to initiate database queries and statistical models; all database activity is logged; only aggregate statistical outputs leave the platform environment following best practice for anonymisation of results such as statistical disclosure control for low cell counts [2].

The OpenSAFELY research platform adheres to the obligations of the UK General Data Protection Regulation (GDPR) and the Data Protection Act 2018. In March 2020, the Secretary of State for Health and Social Care used powers under the UK Health Service (Control of Patient Information) Regulations 2002 (COPI) to require organisations to process confidential patient information for the purposes of protecting public health, providing healthcare services to the public and monitoring and managing the COVID-19 outbreak and incidents of exposure; this sets aside the requirement for patient consent [3]. This was extended in November 2022 for the NHS England OpenSAFELY COVID-19 research platform [4]. In some cases of data sharing, the common law duty of confidence is met using, for example, patient consent or support from the Health Research Authority Confidentiality Advisory Group [5].

Taken together, these provide the legal bases to link patient datasets on the OpenSAFELY platform. GP practices, from which the primary care data are obtained, are required to share relevant health information to support the public health response to the pandemic, and have been informed of the OpenSAFELY analytics platform.

1 NHS Digital. Data Security and Protection Toolkit. 2020. https://digital.nhs.uk/data-and-information/looking-after-information/data-security-and-information-governance/data-security-and-protection-toolkit (accessed 30 April 2020)
2 NHS Digital. ISB1523: Anonymisation Standard for Publishing Health and Social Care Data. https://digital.nhs.uk/data-and-information/information-standards/information-standards-and-data-collections-including-extractions/publications-and-notifications/standards-and-collections/isb1523-anonymisation-standard-for-publishing-health-and-social-care-data (accessed 30 April 2020)
3 Secretary of State for Health and Social Care - UK Government. Coronavirus (COVID-19): notification to organisations to share information. 2020. https://web.archive.org/web/20221101161400/https://www.gov.uk/government/publications/covid-19-notification-to-gps-and-nhs-england-to-share-information (accessed 30 January 2022)
4 Secretary of State for Health and Social Care - UK Government. Coronavirus (COVID-19): notification to organisations to share information. 2022. https://www.gov.uk/government/publications/coronavirus-covid-19-notification-to-organisations-to-share-information/coronavirus-covid-19-notice-under-regulation-34-of-the-health-service-control-of-patient-information-regulations-2002

5 NHS Health Research Authority. Confidentiality Advisory Group. https://www.hra.nhs.uk/about-us/committees-and-services/confidentiality-advisory-group/ (accessed 30 January 2023)
